# Necessity of Re-Vaccination

**Published:** 1854-02

**Authors:** G. Benedict

**Affiliations:** Physician to the North-Western Dispensary, New York


### *On the Necessity of Re-Vaccination.*

BY G. BENEDICT, M. D., PHYSICIAN TO THE NORTH-WESTERN DISPENSARY, NEW YORK.

The complete failure of vaccination in some instances to protect the system against small-pox, and its partial failure in other cases, has led to various theories and practices. Some have, in a measure, doubted its efficiency, others have rejected it as worthless, while others still have endeavored to discover the reason of such failures, in order ifpossible,to obviate them.

Action has wonderfully corresponded with sentiment. Vaccination has been carelessly performed, aud suffered to run its course unregarded, or it has been entirely neglected ; or, on the other hand, has been performed with due care and watched with interest.

As yet the fact stands as it ever has done. Vaccination sometimes fails, sometimes it seems to exert a complete protecting influence against a most loathsome and very fataldisease. Hence the question,as to the cause of this, is in truth a very important one. It will hardly do to put it aside, by considering the case analogous to that in which specific remedies for various disorders are employed with more or less general success, and yet with now and then a failure. For we have no so-called specific for any one disease, which is not also used with more or less benefit in other diseases, so that in different states of the constitution, it is productive of good results. We have yet to learn that the vaccine infection affords the least protection to any other than the variolous disease. It is said by some to exert a protecting influences against measles, by rendering the attack less severe; but our own observation contradicts the assertion. The mode, too, of introducing this protecting agent into the system is different from that of other remedies for existing or expected disease. It is with a view to save the patient from the peril and disfigurement of small-pox that it isemployed, and for this alone; and this object we believe it capable of effecting. If we attempt to account for its apparent failure in some instances, on the ground that there is a greater susceptibility to variola in some individuals than in others, we are met by the fact, that many thousands of those who would undoubtedly have suffered from, and succumbed to, the disease, have been saved; and this, too, when the oper ation of vaccination has been so carelessly performed. If those only who are least liable to the variolous affection are to be benefitted, the great value of the discovery is taken from it. I can conceive no better evidence of the great susceptibility to a disease, and the value of any remedy for it, than I find in the generally admitted fact, that, where once many died, the employment of such remedy has diminished and almost extinguished mortality from such cause. Nor need we, as it seems to me, adopt the opinion, that the changes effected in the system at puberty destroy the hitherto protective power of vaccination. A change it certainly is from childhood to adolescence; but that the organization of the solids or fluids composing the body undergoes any such modification as to render an active agent inert, or *vice versa*, is at best hypothetical. It looks to much like the old whim that vaccination should be repeated once in seven years, because in that period its power had all "run out" of the system. The reason is as good in one case as in the other. Comparatively few adults, who were vaccinated in infancy or childhood, are susceptible of successful vaccination. How does this happen if there is such a change at puberty ?

Some stress has been laid upon the number and appearance of the cicatrices, as enabling us to judge of the efficacy or inefficacy of vaccination. Doubtless they indicate more or less the efficiency of the pustules, but they alone are not to be relied on. Nor should we judge from the local intensity of the pustule, that it is sufficient or otherwise; for in different individuals and in different states of the constitution, there is great variety in this respect.

We are not permitted to look into the human organs so intelligently as to understand how and by what means all its changes are affected. The action of remedies on the secretions, *e. g.* in various conditions of the system, though the results are often visible and marked enough, is by no means capable of being fully comprehended. And when we come to the question before us—how the active existence of one poison is rendered forever impossible by the previous existence of another and different poison—we hesitate for an answer. The results of experience, however, justify the belief that it is so, and we rest upon this belief as a fixed fact.

A peculiarity in my own person, perhaps not remarkably uncommon in others, has led me to attentive thought and careful observation on this subject. I remember to have been vaccinated in childhood several times, before the presence of the virus manifested itself by the formation of a pustule. It did at length happen, and the cicatrix still remains. While at college, a few cases of variola and varioloid appearing among the students, I was again vaccinated, under the impression, that, as seven and even fourteen years had elapsed, I might be subject to small pox if exposed. Here again I received the infection, and had a pustule larger, and, so far as my memory serves me in regard to the first, more intense than that. About four weeks from the time of re-vaccination, and after my arm had entirely recovered from its effect, I again vaccinated with lymph taken from the arm of a fellow student. Again, and so soon after the second vaccination, I had a large pustule, which went through a regular course, the scab adhering until about the twelfth day. Now here after the re-vaccination, I would have been considered as safe as the vaccine disease could render me, and doubtless, had I suffered from variola, my case would have been set down as one of those in which vaccination had availed nothing. And yet was there any reason why I should not have suffered the full force of the disease, had I been exposed ? Since that time I have repeatedly inserted the virus in different situations, with no other effect than the slight irritation which is known to follow the scratch of the lancet charged with the poison in those thoroughly vaccinated. My own experience has been partly confirmed by observation. I have re-vaccinated many children, and quite a number of them those in whom I have watched the progress of the first pustule. I have seen the re-vaccination unequivocally successful in only eight cases, and in no instance have I been satisfied that true vaccinia was present the third time. Re-vaccination of adults has been successful in about the same proportion as in children.

My observations have not been sufficiently extensive to establish any new fact, but I make them known that others may observe also, and see if they do not confirm the following proposition:

*That vaccination, properly performed, and repeated until the susceptibility to the vaccine disease is exhausted from the system, affords entire immunity from the variolous disease.*

It may seem that, by including so much, my propositions is worthless, as it would extinguish not only the genuine disease, but its modification, varioloid. But we are to bear in mind that one, two or three successive pustules may still leave the system unprotected, at least in part. Vaccination should be repeated until nothing like a pustule can be obtained. Let each one obsere for himself, until evidence accumulates which shall sustain or overthrow this position; and let no one say that vaccination is not a protection for those in whom the susceptibility to variola is unusually strong, until he first ascertains whether there is not still left some susceptibility to vaccinia.—*N. Y. Jour. of Medicine.*

